# Timing of Parenteral Nutrition in Neonates, Infants, and Children: Current Evidence, Circadian Considerations, and Emerging Directions

**DOI:** 10.3390/nu18172800

**Published:** 2026-08-27

**Authors:** Alvin P. Chan, Rochelle W. Lai, Kara L. Calkins

**Affiliations:** 1 Division of Gastroenterology, Hepatology and Nutrition, Department of Pediatrics, David Geffen School of Medicine, University of California, Los Angeles (UCLA), Los Angeles, CA 90095, USA; alvinchan@mednet.ucla.edu; 2 Division of Cardiology, Department of Medicine, David Geffen School of Medicine, University of California, Los Angeles (UCLA), Los Angeles, CA 90095, USA; rochellelai@g.ucla.edu; 3 Division of Neonatology & Developmental Biology, Department of Pediatrics, Neonatal Research Center of the UCLA Children’s Discovery and Innovation Institute, David Geffen School of Medicine, University of California, Los Angeles (UCLA), Los Angeles, CA 90095, USA

**Keywords:** parenteral nutrition, lipids, pediatrics, neonate, infant, chrononutrition, circadian

## Abstract

Despite advances in parenteral nutrition (PN) formulations, the temporal aspects of PN delivery are less defined. This review synthesizes evidence on the optimal timing of PN initiation, duration, and delivery within the 24-h day in pediatric populations. Evidence suggests that the optimal timing of PN initiation varies by age and clinical context. While early PN remains standard practice for very preterm and very low birth weight infants, delaying PN has been associated with improved outcomes in critically ill children. Continuous PN is routinely initiated in neonates and critically ill children to minimize fluid and metabolic fluctuations, whereas cyclic PN is generally used in older infants and children who are clinically stable to allow infusion-free periods and greater mobility. Despite the widespread use of overnight PN infusions in both the hospital and home settings, the clinical implications of aligning PN delivery with endogenous circadian rhythms remain very limited. Future studies should establish optimal temporal strategies for different pediatric populations and determine whether optimizing the timing, infusion pattern, and circadian alignment of PN delivery improves growth, development, metabolic health, and quality of life.

## 1. Introduction

**Figure 1:**
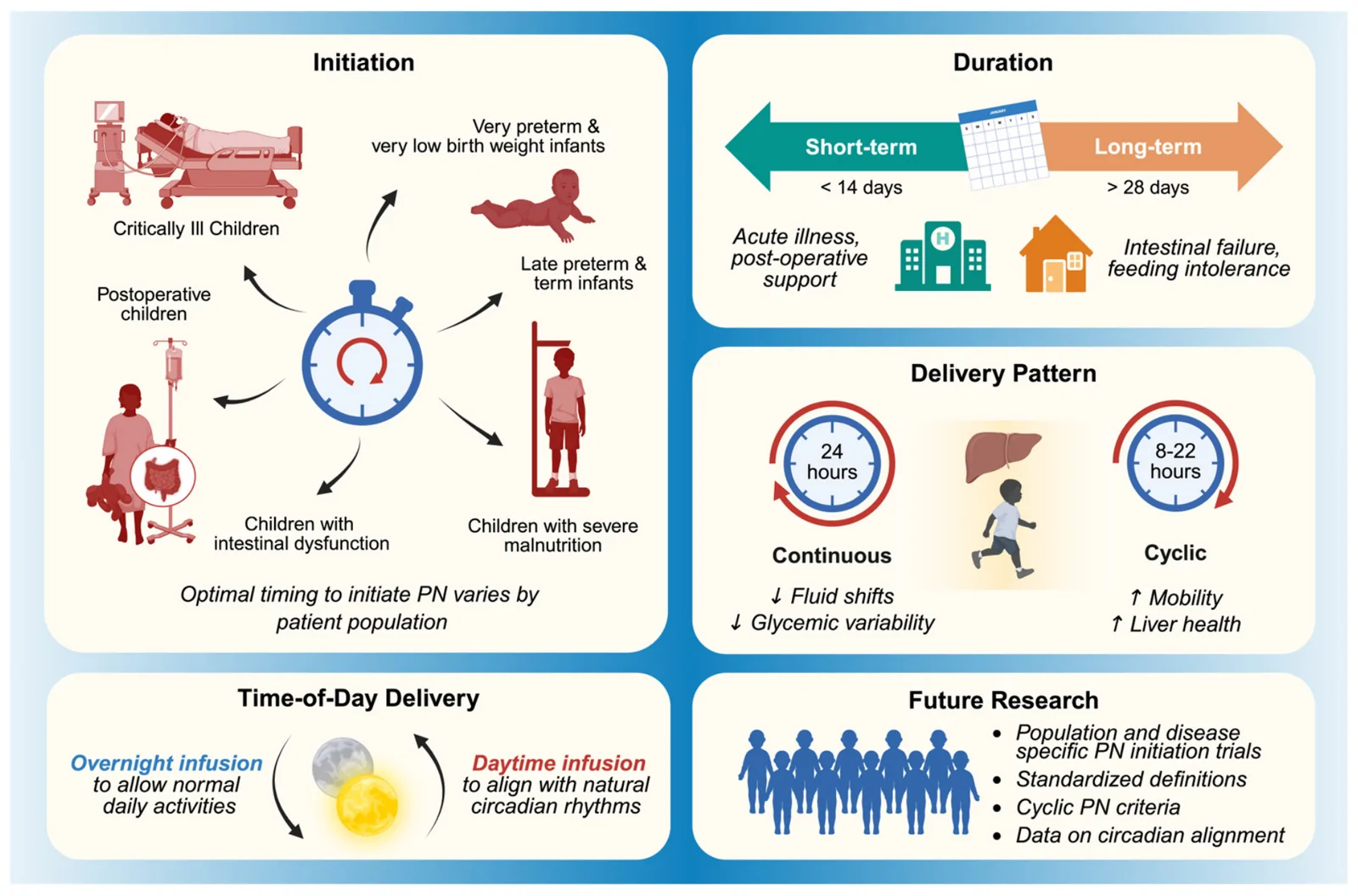
Temporal aspects of PN delivery and key priorities for future research.

Parenteral nutrition (PN) is a life-sustaining therapy for neonates, infants, and children who cannot meet their fluid and nutritional needs enterally [1]. PN supports short- and long-term growth and development, but requires careful management given the risks of nutrient deficiencies and toxicities, metabolic derangements (i.e., hyper- or hypoglycemia, hypertriglyceridemia, and refeeding syndrome), infection, intestinal failure-associated liver disease, and financial costs [2,3]. Research has mainly focused on the composition and doses of macronutrients. However, the temporal aspects of PN delivery, including the timing of initiation, duration, and infusion schedule within the 24-h day, have received comparatively little attention despite their biological and clinical relevance. The timing of PN may influence nutrient metabolism independently of PN composition. Metabolic capacity is influenced by developmental and clinical status, while nutrient utilization and hormonal responses fluctuate across the circadian cycle. Thus, the consideration of when PN is delivered is an important complement to the established focus on what parenteral nutrients are delivered. When considering the optimal timing of PN in pediatric patients, clinicians must balance the risk of dehydration and malnutrition with potential complications secondary to PN. In this review, we evaluate the evidence surrounding four key temporal aspects of PN delivery: (1) timing of initiation across different pediatric populations, (2) duration of PN therapy, (3) delivery pattern, and (4) timing of delivery within the 24-h circadian cycle. Figure 1 provides an overview of these temporal domains.

## 2. Methods

This is a narrative review of the scientific literature to summarize the available evidence on the timing of PN in the pediatric population. The review was based on a structured search of PubMed/MEDLINE and Google Scholar for articles published in English through June 2026, with priority given to more recent literature over the past 10 years. Search terms included combinations of “parenteral nutrition”, “timing”, “duration”, “critical illness”, “malnutrition”, “chrononutrition”, “circadian rhythm”, “pediatric”, “neonate”, “preterm”, and “infant”. Studies were eligible for inclusion if they addressed the temporal aspects of PN administration in neonatal and pediatric populations. Randomized controlled trials and meta-analyses were prioritized, and observational prospective studies were included if they were not available. Expert opinion and consensus statements by relevant professional societies were also referenced to contextualize current practice recommendations. Given the narrative nature of this review, no formal risk-of-bias assessment or quantitative synthesis was performed. Instead, studies were evaluated based on study design, quality of evidence, consistency of findings across studies, and their relevance to informing optimal PN timing in pediatric care.

## 3. Initiation of Parenteral Nutrition

### 3.1. Preterm and Very Low Birth Weight Infants

The timing of PN initiation in neonates varies according to gestational age, birth weight, day of life, underlying gastrointestinal function and pathology, and critical illness. Given their limited nutrient stores and risk for feeding intolerance, very preterm infants (<32 weeks gestational age) and very low birth weight infants (birth weight < 1500 g) rapidly accrue protein and energy deficits in the absence of enteral nutrition. Thus, PN is routinely initiated within hours of birth to provide fluids, electrolytes, macro- and micronutrients to support growth and prevent malnutrition [4,5]. Although PN is considered standard practice in these high-risk infants, the optimal timing of initiation remains uncertain. Interpretation of the available literature is complicated by substantial heterogeneity in study design, including differences in definitions of “early” versus “late” PN, composition of PN, dosing strategies, and advancement protocols.

Several randomized controlled trials have evaluated the effects of early PN initiation in preterm infants, with mixed results. In a small randomized controlled trial of 17 preterm infants (<34 weeks gestational age), initiation of parenteral amino acids at 1.5 g/kg/day within the first 24 h of life, followed by advancement to 2.5 g/kg/day by day of life 3, did not shorten the time required to regain birth weight compared to initiation of parenteral amino acids at 2.5 g/kg/day on day of life 3 [6]. Corroborating these findings, a larger study randomized 135 very low birth weight infants to receive 2.4 g/kg/day of amino acids either at birth or on day of life 3 [7]. While no differences in short-term growth were observed between the two groups, early amino acid supplementation was safe and improved nitrogen balance and albumin synthesis rates [6,7,8]. In a third randomized trial, initiating both amino acids and intravenous lipids shortly after birth was associated with less weight loss and fewer days to regain birth weight compared with delaying PN until day of life 3 [9]. It remains unclear if these results were secondary to the early provision of both parenteral amino acids and intravenous lipids or chance.

Observational studies have also examined the clinical impact of early PN initiation in preterm infants. In a large propensity score-matched observational study of 16,294 very preterm infants, those who received PN in the first 48 h were more likely to survive to hospital discharge (absolute risk difference [ARD] 3.3%). This finding may reflect an improvement in neonatal care or survival bias since the study only included neonates who survived the first 48 h of age. Conversely, infants who received PN after 2 days of life were less likely to develop common co-morbidities associated with prematurity, such as chronic lung disease (ARD 1.24%), treatment-requiring retinopathy of prematurity (ARD 0.50%), and sepsis (ARD 0.84%) [10]. In a follow-up study at approximately 2 years of age, after adjusting for various confounders, males who received early PN were six times more likely to have a “normal outcome,” defined as the absence of a major disability. In contrast, females exhibited a lower mental developmental index [11].

Hence, while most experts, including the European Society of Pediatric Gastroenterology, Hepatology and Nutrition (ESPGHAN) and American Society of Parenteral and Enteral Nutrition (ASPEN), recommend early PN for all very preterm and very low birth weight infants, the evidence supporting its optimal timing remains inconclusive. Studies have generated not only null findings, but have also highlighted a potential concern for harm [4,5,6,7,9,10,11]. Further research in this population is required to understand how PN timing alters mortality and morbidities and which outcomes are driven by specific macronutrients and doses (i.e., amino acids), biologically relevant variables (i.e., sex, gestational age, birth weight), and severity of illness and inflammation.

### 3.2. Late Preterm and Term Infants

In late preterm (34–37 weeks gestational age) and term neonates (>37 weeks gestational age), decisions regarding PN initiation can be broadly separated into two categories: immediate initiation (within hours of birth) and delayed initiation (typically within 1–3 days). Immediate PN is indicated in infants for whom enteral nutrition is contraindicated or expected to be delayed (>3–5 days). This includes infants with prenatally diagnosed or immediately apparent gastrointestinal conditions, such as congenital diaphragmatic hernia, intestinal atresia, gastroschisis, and omphalocele. Meanwhile, conditions that are not recognized at birth or develop later in the neonatal course, including necrotizing enterocolitis, spontaneous intestinal perforation, and gastrointestinal obstruction, will generally necessitate PN initiation once diagnosed. In addition, critically ill infants with conditions associated with impaired gastrointestinal perfusion, such as critical congenital heart disease, shock, or the use of high-dose vasoactive medications, may prompt immediate PN initiation [2].

In contrast to very preterm infants, late preterm and term infants generally have greater nutritional reserves and may not require immediate PN initiation following admission to the neonatal intensive care unit after birth. Clinical judgment is required to determine if and when PN should be initiated [2,3]. In all cases, the timing, extent (supplemental versus total support), and volume of PN are guided by hemodynamic stability, kidney function, severity of disease, anticipated fasting duration, and enteral feeding tolerance.

Given the heterogeneity among late preterm and term infants, it has been difficult to produce generalizable evidence to guide optimal timing of PN initiation in this population. As a result, consensus guidelines vary. In stable late preterm and term infants, enteral feeding, if safe, should always be prioritized. ESPGHAN guidelines recommend that PN should be initiated after 48–72 h if enteral nutrition is not feasible in critically ill term infants [12]. The National Institute for Health and Care Excellence (NICE) guidelines recommend initiating PN within 72 h of birth if enteral feeding cannot be established for late preterm and term infants. Further, NICE guidelines specify that central venous delivery of PN should be prioritized, whereas peripheral PN should be considered only if it avoids the delay of PN initiation or only short-term PN use (<5 days) is anticipated [13].

Few studies have specifically investigated the timing of PN in late preterm and term infants. A multisite retrospective cohort study evaluated two matched cohorts for clinical outcomes (13,712 infants) and mortality (21,402 infants) [14]. These late preterm and term infants received either early (within the first 24 h of life) or late (after the first 24 h to day of life 10). Propensity matching between groups controlled for important biologically and clinically relevant covariates, such as sex, race, gestational age, birth weight, 5-min Apgar score, congenital anomalies, and respiratory support and treatment. When the group who received early PN was compared to the group who received late PN, there were no statistically significant differences for median length of stay (11 days versus 11 days), median mechanical ventilation days (conventional: 3 days versus 3 days; high frequency: 5 days versus 4 days), or occurrence of late-onset sepsis (38 cases versus 51 cases). Likewise, there was no difference in deaths (61 cases versus 56 cases). Secondary analysis in the late PN group revealed an association between delaying PN initiation and hospital length of stay. Compared to initiation on day of life 0, PN on days of life 4–10 was associated with a longer hospital stay (1.16 rate ratio [RR]; 95% confidence interval [CI] 1.14–1.18), followed by day of life 3 (0.98 RR; 95% CI 0.97–0.99), day of life 2 (0.96 RR; 95% CI 0.95–0.97), and day of life 1 (0.96 RR; 95% CI 0.95–0.96). However, the infants whose PN was initiated on days of life 4–10 most likely required prolonged hospital stays due to their underlying clinical status, including the need for prolonged mechanical ventilation and higher rates of late-onset sepsis. Confirming these findings, the infants whose PN was initiated on days of life 4–10 demonstrated greater rates of mortality (1.83 RR; 95% CI 1.33–2.52). The generalizability of this study is limited considering that “early” PN was defined as initiation within 24 h of life, while “late” PN spanned several days (day of life 1–10).

In another study, 60 late preterm and term infants (≥34 weeks gestational age; ≤28 days of age) who were receiving ≤ 50% of their intake as enteral nutrition were randomized to either early PN (initiated on days 1–2 of admission) or late PN (initiated on day 6 of admission). The study was not powered for a specific clinical outcome. Instead, it was powered to detect differences in F_2_ isoprostanes, a marker of lipid peroxidation, and phenylalanine, an essential amino acid. The postnatal age at randomization was comparable between groups, with a mean of 2.3 days. Approximately 80% of the participants were term infants. While PN formulations varied according to the infant’s clinical status and hospital guidelines, all infants in the early PN group received amino acids and lipids, whereas not all infants in the late PN group received both macronutrients. In the early PN group, infants demonstrated a smaller mean decline in weight z-scores (baseline to hospital discharge) (−0.6 z-scores [0.6 standard deviation (SD)] versus −1.0 z-scores [0.6 SD]). Despite improved growth, the severity of postnatal growth restriction was comparable between the early and late PN groups (mild: 23.3% versus 33.3%; moderate: 13.3% versus 20%; normal: 63.3% versus 43.3%; severe 0% versus 3.3%). Moreover, infants in the early PN group displayed a significantly higher incidence of hyperglycemia (66.7% versus 36.7%); there was no difference in hypoglycemia. Other outcomes, such as length of stay (23 days versus 19 days), hospital-acquired infections (8 cases versus 3 cases), and time to reach full enteral feeds (12 days versus 9 days) were modestly higher in the early PN group, although not statistically significant. There were no differences in plasma F_2_-isoprostane at any time point between the two groups, suggesting that the timing of PN initiation does not affect the degree of oxidative stress [15].

A retrospective study examined the outcomes of 185 infants ≥ 35 weeks gestational age who received PN or dextrose-containing intravenous fluids within the first 36 h of life. It is important to note that the PN composition was not clearly defined in this study. All infants in this cohort were admitted to the neonatal intensive care unit for hypoxic–ischemic encephalopathy and received therapeutic hypothermia. The infants who received PN exhibited a statistically significant lower incidence of hypoglycemia and acute kidney injury. Other outcomes, such as hypoxic–ischemic encephalopathy severity, length of stay, time to reach full enteral feeds, days with a central line, and bloodstream infection rates were comparable between groups. Growth was reported as a percent weight difference at day of life 6, with no significant difference (−0.6% versus −0.9%) [16]. Although this study is limited to a specific clinical condition, there appears to be fewer complications in infants with hypoxic–ischemic encephalopathy who received therapeutic hypothermia who were prescribed early PN, compared to infants who were prescribed intravenous dextrose fluids.

Taking these studies together, it appears beneficial to initiate PN within the first 1–3 days of life and to avoid prolonged PN delays (>4–10 days) in late preterm and term infants if enteral nutrition is not anticipated or limited. This general guideline is supported by consensus statements from ESPGHAN and NICE [12,13]. Further research using properly powered randomized controlled trials is warranted to explore the direct effects of PN initiation timing on clinically relevant outcomes, like length of stay, sepsis, days of respiratory support, growth, glycemic control, and mortality. Future studies should also utilize more discriminate timeframes for early versus late PN and clearly stratify co-morbidities, medical acuity, and specific diagnoses.

### 3.3. Postoperative Infants and Children

Postoperative infants and children represent a distinct population in whom the need for PN is often anticipated, given predictable delays in enteral feeding and increased metabolic demands after surgery. Postoperative metabolic needs are dynamic and vary between the early acute (inflammatory response, need for cardiorespiratory support), late acute (stabilized clinical symptoms, reduced cardiorespiratory support), and recovery or stable (no cardiorespiratory support required, anabolic protein metabolism) phases [12]. There are no well-powered studies that address the estimated needs of postoperative infants recovering from surgery. Consensus guidelines from the ESPGHAN, European Society for Clinical Nutrition and Metabolism (ESPEN), European Society for Paediatric Research (ESPR), and Chinese Society for Parenteral and Enteral Nutrition (CSPEN) recommend that energy intake in the acute phases of critical illness, not specific to etiology, is equal to or lower than measured energy expenditure [17]. However, once the inflammatory response resolves, energy intake should increase to approximately 1.3 times the resting energy expenditure to support anabolism and growth in the recovery phase [12,17]. This equates to 90–120 kcal/kg/day for preterm and 75–85 kcal/kg/day for term infants [17]. Indirect calorimetry for infants recovering from corrective surgery for congenital gastrointestinal anomalies showed that preterm infants with resting energy expenditures of 55.7–67.4 kcal/kg/day and energy intakes of 80.7–109.4 kcal/kg/day achieved adequate weight gain, while term infants with similar energy expenditures (57.3–67.9 kcal/kg/day) but lower intakes (74–105.2 kcal/kg/day) exhibited slower weight gain [18]. Hence, infants who undergo major gastrointestinal surgery require higher energy intakes to promote growth in the recovery phase. PN support can help meet these increased energy requirements; however, there are no precise recommendations on when to start PN postoperatively, particularly in the early acute phase for infants recovering from gastrointestinal surgery [19,20]. Infants with critical congenital heart disease may not be able to achieve full enteral nutrition prior to palliative or corrective surgery and may experience prolonged fasting periods postoperatively. In these populations, consensus guidelines for energy intakes in the different phases of critical illness, particularly after surgery, may also apply [17].

Another important consideration following gastrointestinal surgery is when enteral nutrition should be initiated, how PN should be weaned, and when PN should be discontinued. Enteral nutrition should be initiated as soon as safely possible after bowel surgery to promote intestinal recovery and minimize PN exposure. Early enteral feeding is an important component of recovery. A large meta-analysis of 1286 children across 10 studies demonstrated earlier achievement of full enteral feeds, shorter hospital stays, and lower rates of infection, without an increased risk of anastomotic complications when feeding was initiated within 48 h compared with later initiation of enteral nutrition [21]. However, the exact timing and rate of enteral advancement should be individualized according to the underlying diagnosis, specific co-morbidities, type of bowel surgery, return of bowel function, and feeding tolerance. Mechanistically, enteral nutrition provides luminal stimulation that promotes intestinal adaptation and facilitates weaning from PN [22].

In summary, PN is commonly initiated early in the postoperative course when enteral nutrition is not expected to meet energy requirements for several days. Ultimately, the precise timing and parenteral calories for each patient should be individualized and guided by clinical judgment and the specific post-operative phase. Further research is warranted to better understand how PN timing alters operative outcomes for pediatric patients.

### 3.4. Critically Ill Children

In the pediatric critically ill patient (>1 month to <18 years), PN initiation depends on numerous factors, including disease state, hemodynamic stability, degree of malnutrition, and prognosis. Enteral feeding should always be prioritized if safe, since enteral nutrient delivery promotes gastrointestinal integrity and motility. PN should be initiated only as a supplement or as the sole source of nutrition when enteral feeds are contraindicated. ASPEN and Society of Critical Care Medicine guidelines recommends that PN not be initiated within 24 h of pediatric intensive care unit (PICU) admission [23], while ESPGHAN states that withholding PN for the first week of PICU admission (with the exception of vitamin supplementation) can be considered [17], based on the Early versus Late Parenteral Nutrition in the Pediatric Intensive Care Unit (PEPaNIC) trial. This multicenter, prospective, controlled trial enrolled 1440 PICU patients, including neonates and adolescent children up to 17 years of age. Participants were randomized to either early (within 24 h of admission) or late (on the eighth PICU day) PN to supplement enteral feeding. Patients who received late PN therapy had statistically significant shorter PICU stays (mean 6.5 days ± 0.4 standard error [SE] versus 9.2 days ± 0.8 SE), decreased occurrence of new infections (10.7% versus 18.5%), fewer days on mechanical ventilation (4.4 days ± 0.3 SE versus 6.4 days ± 0.7 SE), and a decreased incidence of renal insufficiency requiring dialysis (2.5% versus 3.6%) [24]. In a parallel adult version of this study, Impact of Early Parenteral Nutrition completing enteral Nutrition In Critical illness (EPaNIC) trial, a cost analysis of this randomized controlled multicenter trial revealed that early PN led to increased financial expenditures due to the cost of PN and anti-infective agents [25].

A subgroup analyses of term infants in the PEPaNIC trial showed comparable findings; however the infants who received late PN were at higher risk of hypoglycemia [26,27]. While these results are intriguing, these findings may not be directly translatable to the neonatal intensive care unit, where most neonates are admitted shortly after birth. In contrast, in the pediatric intensive care unit, most neonates are admitted from home in the first couple weeks of life. Moreover, the study was not powered for any specific sub-group analysis. The authors performed follow-up studies on long-term growth, neurocognitive function, and physical development in the larger cohort of 1440 participants after 2 and 4 years of age. After two years, only 55% of the initial cohort was available for assessment, with most metrics obtained by parent/caregiver report. When the late PN group was compared to the early PN group, the late PN group demonstrated better executive functioning (−2.258 β estimate [βE]; 95% CI −4.012 to −0.504; lower scores represent better performance), fewer externalizing behavioral problems (−1.715 βE; 95% CI −3.325 to −0.106), and improved visual-motor integration (0.468 βE; 95% CI 0.087–0.850) [28]. The four-year follow-up study revealed comparable results; however, only 48% of the initial cohort participated. The parents/caregivers in the late PN group reported fewer internalizing (−1.88 βE; 95% CI −3.69 to −0.07), externalizing (−1.73 βE; 95% CI −3.43 to −0.03), and total emotional and behavioral problems (−2.44 βE; 95% CI −4.22 to −0.67) compared to the early PN group [29].

There are some limitations to the PEPaNIC trial. Clinical outcome definitions were not always clearly defined. For example, the diagnostic criteria for newly acquired infections, a primary endpoint, were not standardized. There was also no information on how many individuals received central or peripheral PN delivery, which may contribute to infection risk. In an ESPGHAN/ESPEN/ESPR/CSPEN position paper, the authors highlighted that some children who received PN may not have needed PN, and that the study may not represent children with chronic diseases or severe malnutrition. Hence, the early PN group may have been overfed [17].

Overfeeding in critical illness, especially when on mechanical ventilation, can lead to complications [30] and could explain why the early PN group exhibited worse outcomes. Early PN can suppress beneficial fasting pathways, like autophagy and ketogenesis, and alter thyroid hormone metabolism, which supports muscle regeneration and helps resolve inflammation. In addition, early PN could have promoted feeding-resistant catabolism and metabolic damage by increasing urea cycle activity leading to anabolic resistance, iatrogenic hyperglycemia resulting in mitochondrial damage, and dysregulated DNA methylation and telomere shortening [31]. These explanations are theoretical and deserve further investigation.

Nonetheless, as a general strategy, current guidelines recommend that PN not be initiated immediately upon PICU admission and should be delayed for at least 24 h. The threshold for and timing of PN initiation should be individualized and clinicians should account for age, nutritional status, disease severity, co-morbidities, and ability to tolerate enteral nutrition [23].

### 3.5. Children with Severe Malnutrition

The optimal timing of PN initiation in children with severe malnutrition, defined as weight-for-length z-score < −3 or body-mass-index (BMI) z-score < −3 [32] based on a single timepoint, is particularly challenging, as clinicians must balance the need to restore nutritional deficits against the potential risks of early nutrient delivery. In the randomized controlled trial, PEPaNIC [24], children at risk for malnutrition were identified at enrollment using the Screening Tool for Risk on Nutritional Status and Growth (STRONGkids) [33]. A subsequent study included a subgroup analysis of 289 malnourished children (mean weight z-score < −3) and found that those who received late PN were less likely to develop new infections (11.3% versus 22.3%), had shorter PICU stays (4 days versus 6 days), and required fewer days of mechanical ventilation (2.5 days versus 3 days) [34]. These findings suggest that pre-existing malnutrition alone may not justify early PN initiation in critically ill children. However, the results should be interpreted cautiously since nutritional status was determined using weight alone at a single timepoint, which may not accurately reflect nutritional stores due to fluid alterations during critical illness. In addition, this was a subgroup analysis and, hence, not appropriately powered for these specific outcomes.

Beyond the question of when to initiate PN, infants and children with severe malnutrition are also particularly vulnerable to complications when nutrition is reintroduced. The initiation of any nutritional support, including PN, should be prescribed with caution given the risk of refeeding syndrome, a constellation of metabolic and electrolyte abnormalities that occur when nutrition is reintroduced or increased after a prolonged period of nutrient deprivation. Under normal physiologic conditions, the intake of nutrients, like glucose, elicits postprandial insulin secretion to promote metabolic processes like glycolysis and oxidative phosphorylation. The molecular machinery for these processes requires cofactors such as potassium, phosphorus, magnesium, and thiamin that normally redistribute from systemic circulation into cells, without clinical consequence. However, during a prolonged nutritive deficit, total body stores of these micronutrients are depleted. Upon refeeding, the sudden reintroduction of nutrients triggers an insulin surge that leads to rapid intracellular shifts of potassium, phosphorus, magnesium, and thiamin, diverting them away from critical physiologic functions, such as nerve transmission and muscle contraction. This abrupt reallocation of electrolytes can precipitate severe complications, including potentially lethal cardiac arrhythmias [35,36].

ASPEN has developed guidelines for accurate screening and treatment of refeeding syndrome in pediatric patients (ages 28 days to 18 years). To identify the risk for refeeding syndrome, providers must assess anthropometric data (weight-for-length or BMI, weight loss, mid-upper arm circumference), energy intake, comorbidities, and serum electrolyte concentrations. Populations at high risk for refeeding syndrome may include individuals with anorexia nervosa, mental health disorders, substance-use disorders, bowel resections, chronic malabsorption, renal failure, critical illness, or prolonged inadequate intake due to starvation or child abuse. In the pediatric population, meeting 3 of 6 mild risk criteria, 2 of 7 moderate risk criteria, or 1 of 7 significant risk criteria prompts surveillance for refeeding syndrome. Once the risk for refeeding syndrome has been determined, nutrition must be gradually advanced. Depending on feasibility, enteral, parenteral, or both routes may be used. Regardless of route, nutrition should be initiated in pediatric patients at a glucose infusion rate of 4–6 mg/kg/minute, advancing by 1–2 mg/kg/minute, until a maximum of 14–18 mg/kg/minute. Notably, ASPEN does not provide recommendations for prophylactic electrolyte supplementation for neonates and children at risk for refeeding syndrome due to a lack of evidence [36]. For adults at risk for refeeding syndrome, prophylactic electrolyte supplementation is recommended unless plasma concentrations are elevated [37]. Thiamine supplementation should be provided to all children at risk for refeeding syndrome prior to and during the initiation of nutrition, given its essential role in glucose metabolism [36].

Emerging evidence suggests that the ASPEN recommendations may not fully address the unique risk of refeeding syndrome in preterm neonates. In the multicenter, double-blind, randomized, placebo-controlled Protein Intravenous Nutrition on Development (ProVIDe) trial, 434 extremely low birth weight infants (birth weight < 1000 g) were randomly assigned to receive either an extra 1 g/day of parenteral amino acids or placebo for the first 5 days after birth, in addition to standard nutritional management. The primary outcome was survival free from neurodevelopmental disability at 2 years corrected age. In a post hoc analysis, refeeding syndrome, defined as serum phosphate < 1.4 mmol/L and total calcium > 2.8 mmol/L, occurred significantly more frequently among infants receiving additional amino acids compared with placebo (24.4% versus 15.7%; adjusted relative risk 1.64; 95% CI 1.09–2.47). The investigators proposed that refeeding syndrome may have contributed to the increased risk for neurodevelopmental impairment observed in the group that received additional amino acids [38]. Although early PN is generally recommended to support growth in preterm infants, these findings suggest that aggressive early amino acid administration may increase the risk of refeeding syndrome in extremely low birth weight infants and preterm infants with fetal growth restriction. However, it remains unknown whether altering the timing of PN initiation, limiting the dose of amino acids, and gradually advancing the dose of amino acids in these high-risk infants could reduce the incidence of refeeding syndrome while preserving the benefits of early nutritional support. Further investigation is needed to determine whether tailored PN timing and dose escalation strategies can mitigate refeeding syndrome in this population.

Across pediatric populations, the optimal timing of PN initiation depends on prematurity, nutritional status, underlying disease, and the risks associated with delaying or initiating nutrition. There remains a lack of robust randomized controlled clinical trials to determine best practices for the timing of PN within individual pediatric subgroups. A Table 1 provides broad recommendations for PN initiation in specific pediatric populations. Future studies should consider whether early or late initiation of individual components of PN, such as amino acids or lipids, along with specific dose escalation strategies, alters clinical outcomes.

## 4. Duration of Parenteral Nutrition Initiation

### 4.1. Short- Versus Long-Term Parenteral Nutrition

Once PN is initiated, the duration of therapy varies among neonates, infants, and children depending on the underlying clinical context. Short-term PN (typically <14 days) is frequently used to provide temporary nutritional support while enteral feeding is being established. Common clinical scenarios include transient feeding intolerance and temporary contraindications to enteral feeding (i.e., post-surgery, hemodynamic instability, gastrointestinal bleeding, bowel ischemia, intestinal obstruction, or prolonged ileus) [39].

In contrast, some extremely low birth weight and extremely preterm infants (gestational age < 28 weeks) require PN for 14–28 days. Infants and children with intestinal failure require long-term PN (>28 days). Intestinal failure is defined as a PN requirement of at least 60 days within a 74-day consecutive day period [40]. The primary gastrointestinal indications for long-term PN are short bowel syndrome secondary to surgical necrotizing enterocolitis, intestinal atresia, and malrotation with volvulus, and less commonly, mucosal enteropathies and severe motility disorders [40]. Although PN is essential for infants and children with intestinal failure, prolonged PN carries significant risks, including central line-associated bloodstream infections, thromboses, metabolic bone disease, lipid and glycemic abnormalities, and intestinal failure-associated liver disease, a progressive cholestatic disorder characterized by hepatobiliary dysfunction in the setting of prolonged PN [41,42,43]. Importantly, PN duration is linked to the underlying disease and ability to achieve enteral autonomy; therefore, associations between prolonged PN exposure and adverse outcomes may reflect both the cumulative effects of PN and the clinical conditions necessitating its continued use. Advances in intestinal rehabilitation programs, rigorous prevention of central line-associated bloodstream infections, and careful attention to intravenous lipid emulsion dose and composition have vastly improved PN-related complications [40]; however, the timing of PN administration may also play an important role in determining outcomes in this population.

### 4.2. Continuous Versus Cyclic Delivery of Parenteral Nutrition

PN can be delivered as a continuous or cyclic infusion. In neonates, continuous 24-h infusion is standard practice to minimize abrupt fluid shifts and metabolic fluctuations, given their limited glycogen and increased risk of hypoglycemia. Continuous PN is also commonly used in critically ill children, who are particularly vulnerable to hyperglycemia and hypertriglyceridemia [44,45,46,47,48]. However, when used over extended periods, continuous PN can contribute to metabolic dysregulation and hepatobiliary injury. From a metabolic standpoint, continuous PN provides uninterrupted dextrose delivery, leading to sustained insulin secretion. Chronically elevated insulin suppresses adipose tissue lipolysis, limiting the mobilization of essential fatty acids from adipose depots to peripheral tissues [49]. At the same time, persistent insulin signaling promotes a lipogenic hepatic milieu, in which excess dextrose is converted to fat through de novo lipogenesis, contributing to hepatic steatosis and inflammation [50].

Given these risks, PN should be compressed into shorter infusion periods whenever possible. Cycling PN, introduced in the 1970s to improve quality of life and limit risks associated with continuous PN, shortens the daily infusion window and provides a defined fasting period free of intravenous therapy [51]. Compared with continuous PN, cyclic PN more closely mimics physiologic feeding by introducing daily fasting intervals that restore normal oscillations in nutrient uptake, hormone secretion, and metabolic signaling [52,53]. The off-infusion period allows insulin levels to fall and fatty acids to mobilize from adipose tissue, helping preserve essential fatty acid availability and attenuate hepatic lipid loading [49]. Early studies in rodents also suggested that cyclic PN better preserved visceral protein synthesis by improving the transcriptional regulation of albumin [54], although these findings have not directly translated into clinical benefit.

However, cyclic PN should be avoided in preterm infants because of their limited glycogen stores, high fluid requirements, and increased risk of hypoglycemia with shorter infusion durations. As a result, data on cyclic PN in this population is limited. To our knowledge, there is only one randomized controlled trial directly comparing cyclic versus continuous PN in preterm infants [55]. In this study, 70 VLBW infants were randomized to early prophylactic cycling (20 h on, 4 h off) or continuous PN. Although there was no difference in the incidence of PN-associated cholestasis (32% vs. 31%), defined as a serum direct bilirubin > 2 mg/dL, dextrose was infused continuously over 24 h in both groups. Thus, the cyclic intervention did not provide a true nutrient-free fasting interval and the risk for hypoglycemia with PN cycling was not assessed.

As infants mature, the metabolic risks associated with PN cycling decrease. Older infants, including those born preterm, and children who are clinically stable and have reached an appropriate weight can generally tolerate cyclic PN. Transition from continuous to cyclic PN should occur gradually, with consideration of clinical stability, risk for hypoglycemia, and fluid balance [56]. Most older children can tolerate PN compression by 2 to 4 h each day until a target goal of 8 to 12 h is achieved [57], whereas younger infants may only tolerate compression to 20 h because of the risk of hypoglycemia related to limited glycogen stores, rebound hyperinsulinemia, and immature counter-regulatory hormone mechanisms [45].

Although there is no consensus regarding the optimal age or weight at which to initiate cyclic PN, it is generally introduced only after infants are clinically stable and have achieved adequate growth. In a single-center retrospective study evaluating prophylactic cyclic PN for the prevention of PN-associated liver disease in surgical neonates, cyclic PN was initiated at approximately 4 weeks of age in clinically stable infants with an average postmenstrual age > 37 weeks and a body weight ≥ 2.5 kg [58].

### 4.3. Timing of Parenteral Nutrition Within the Circadian Cycle

In addition to how long PN is delivered, when cyclic PN is administered within the 24-h day may have important metabolic consequences. It is generally standard practice for patients receiving home PN support to administer infusions overnight to coincide with sleep, a schedule that offers practical advantages by allowing greater mobility and participation in daytime activities without being tethered to infusion equipment [59,60]. However, this practice has largely stemmed from logistical convenience rather than biological rationale or clinical evidence. Therefore, despite the widespread use of overnight PN, there is a striking paucity of clinical studies comparing nighttime versus daytime PN administration.

From a physiological perspective, concentrating nutrient delivery during the night introduces a potential mismatch with endogenous circadian regulation. The circadian system coordinates daily rhythms in metabolism through a central pacemaker and peripheral clocks in metabolic tissues, including the liver, skeletal muscle, pancreas, adipose tissue, and gastrointestinal tract [61,62]. Circadian disruption, whether through exposure to artificial light or altered feeding schedules, can desynchronize central and peripheral clocks and impair metabolic homeostasis [63,64]. Persistent circadian disruption is increasingly recognized as a risk factor for insulin resistance, hepatic steatosis, and cardiometabolic disease [64]. Consistent with this, a recent American Heart Association Scientific Statement recognized the timing of food intake, or chrononutrition, as an important synchronizer of circadian rhythms and highlighted appropriately timed meals as a potential strategy to promote cardiometabolic health [65]. Whether these principles of chrononutrition extend to the timing of PN delivery remains unknown.

In theory, nocturnal PN could disrupt endogenous circadian programs, but its effects on growth and metabolic outcomes remain largely unknown, particularly in pediatric populations, where no circadian PN studies exist. In a small adult study, five of six patients with dementia receiving overnight PN showed abnormal oscillations of peripheral clock genes (*PER3*, *REV-ERBα*, *REV-ERBβ*) in hair follicle cells, whereas age-matched controls not receiving PN maintained normal rhythmicity [66]. Although limited in scope, this study provides preliminary evidence that nocturnal PN may be associated with altered peripheral circadian rhythms in humans. In rodents, the effect is more pronounced. Rats receiving PN during their active (nocturnal) phase maintained normal rhythmic expression of peripheral clock genes (*Dbp*, *Per2*), whereas infusion during the inactive (light) phase inverted these rhythms [67]. However, whether these circadian perturbations translate into clinically meaningful outcomes remains to be seen.

To date, only one study has examined whether PN timing translates into patient outcomes. In a cohort of 73 adults receiving home PN, 86% administered infusions overnight, with median start and end times of 21:00 and 08:00 [68]. Compared with patients receiving daytime infusions, those receiving overnight PN reported significantly greater sleep disruption and nocturnal urination. Notably, 32% of patients reported that nocturnal nutrition support always disrupted their sleep, and 64% reported sleep disruption due to nocturnal urination. In a separate study, untargeted plasma metabolomics was performed in nine adult patients with short bowel syndrome after one week of overnight home PN and again following one week of daytime PN. Daytime infusions were associated with differences in circulating lipid and amino acid metabolites, with pathway enrichment analysis identifying three pathways related to daytime infusions, including biosynthesis of unsaturated fatty acids, d-arginine, and d-ornithine metabolism. However, the biological significance of these findings is unclear.

Given the metabolic consequences of persistent circadian disruption, dietary strategies aimed at realigning food intake with endogenous circadian rhythms have gained substantial interest. Among these, time-restricted eating, a form of intermittent fasting that consolidates food intake into a defined daily window, has emerged as an effective intervention to improve metabolic health [69]. Together, these findings provide a biological rationale for investigating whether PN delivery aligned with the normal wake cycle may confer similar benefits.

At present, there is no explicit medical guidance regarding the optimal timing of PN delivery [70]. In hospitalized patients, operational factors, including electronic health record workflows, morning laboratory schedules, and staffing logistics, often favor overnight infusions. Home PN, however, may allow greater flexibility in infusion timing, which can be tailored to support daytime schedules, sleep quality, and overall well-being. This flexibility underscores the importance of shared decision-making between clinicians and patients when determining infusion timing.

In summary, accumulating evidence suggests that the time-of-day delivery of PN has biological and physiological relevance. However, the clinical implications of daytime versus overnight PN remain largely untested, especially in the pediatric population. Determining whether adjusting PN timing can improve metabolic outcomes and long-term health represents an important unanswered question. As the field moves toward more personalized approaches to nutrition support, incorporating chrononutrition into PN management may be a promising yet underexplored opportunity, particularly for infants and children requiring lifelong PN.

## 5. Conclusions

PN is a life-sustaining therapy for neonates, infants, and children who cannot adequately meet their nutritional requirements through the gastrointestinal tract. Because nutrition is essential for growth, development, and survival, early PN support is a top priority when full enteral feeding is not feasible. However, accumulating evidence suggests that the optimal timing of PN initiation is more nuanced than previously appreciated. The benefits and risks of early PN vary according to gestational age, birth weight, postnatal age, nutritional reserve, underlying disease, and clinical condition. While early PN remains standard practice for very preterm and very low birth weight infants, the evidence supporting this practice is not consistent. The optimal timing for PN initiation for late preterm and term infants is not well defined; however, experts recommend delaying PN for 48–72 h after birth for most late preterm and term neonates. Even within the extremely low birth weight population for whom early PN is recommended, higher early amino acid intake has been associated with an increased risk of refeeding syndrome and less favorable neurodevelopmental outcomes. This suggests that the implementation of early PN, including the timing and dosing of individual macronutrients, may warrant further refinement. In critically ill children, delaying PN has been associated with improved short- and long-term clinical outcomes, including decreased mortality rates and improved neurobehavioral outcomes. Collectively, these findings demonstrate that the optimal timing of PN initiation depends on the clinical context.

Far less is known about other temporal aspects of PN delivery. While continuous PN is routinely used in neonates and critically ill children, cyclic PN is generally reserved for older infants and children requiring prolonged PN; however, high-quality pediatric evidence comparing these infusion strategies remains limited. Likewise, despite the widespread practice of overnight PN administration in both the hospital and home settings, there is remarkably little evidence to determine whether aligning PN delivery with natural circadian rhythms improves clinically meaningful outcomes.

Future research should consider a “more precise approach” and define the optimal timing of PN across specific pediatric populations and disease states (including periods of acute inflammation and immediate recovery from surgery). Adequately powered multicenter neonatal and pediatric randomized controlled trials with standardized definitions and timing windows are needed to determine which neonates and children benefit from early versus delayed PN initiation and whether individual nutrient components (i.e., amino acids, lipids, and dextrose) should be introduced or advanced at different rates. Trials should incorporate, when feasible, translational studies such as epigenetics, metabolomics, and continuous glucose monitoring. Additional studies should identify which clinical characteristics best predict safe initiation of cyclic PN as well as the transition from continuous to cyclic infusion. As chrononutrition continues to emerge as an important determinant of metabolic health, prospective studies are needed to determine whether aligning PN administration with endogenous circadian rhythms improves sleep quality, metabolic homeostasis, growth, neurodevelopment, and quality of life. Figure 1 summarizes these key directions for future research. In conclusion, optimizing the timing of PN delivery —including initiation, duration, infusion pattern, and circadian alignment— represents an important area for future investigation.

## Figures and Tables

**Table 1:** Recommended Timing of PN Initiation by Population.

Population	Timing of PN Initiation
Very low birth weight infants	Immediately after birth [4,39]
Late preterm & term infants	Within 1–3 days of birth [13,39]
Children & adolescents	Within 4–5 days
Critically ill children	Delay for at least 24 h [23]

## Data Availability

No new data were created or analyzed in this study. Data supporting the findings of this review are available within the cited literature.

## Abbreviations

The following abbreviations are used in this manuscript:

ARD	Absolute risk difference
BMI	Body mass index
CI	Confidence interval
PICU	Pediatric intensive care unit
PN	Parenteral nutrition
RR	Rate ratio
SD	Standard deviation
SE	Standard error
